# Sensors for optical thermometry based on luminescence from layered YVO_4_: Ln^3+^ (Ln = Nd, Sm, Eu, Dy, Ho, Er, Tm, Yb) thin films made by atomic layer deposition

**DOI:** 10.1038/s41598-019-46694-8

**Published:** 2019-07-15

**Authors:** Michael N. Getz, Ola Nilsen, Per-Anders Hansen

**Affiliations:** Department of Chemistry, Centre for Materials Science and Nanotechnology, University of Oslo, Sem Sælands Vei, Oslo, 0371 Norway

**Keywords:** Sensors and biosensors, Optical materials and structures, Optical materials, Sensors, Imaging and sensing

## Abstract

Below the Earth’s crust, temperatures may reach beyond 600 K, impeding the batteries used to power conventional thermometers. Fluorescence intensity ratio based temperature probes can be used with optical fibers that can withstand these conditions. However, the probes tend to exhibit narrow operating ranges and poor sensitivity above 400 K. In this study, we have investigated single and dual layered YVO_4_: Ln^3+^ (Ln = Nd, Sm, Eu, Dy, Ho, Er, Tm, Yb) thin films (100–150 nm) for use in fluorescence intensity ratio based temperature sensors in the 300–850 K range. The type of lanthanide emission can be fine-tuned by adjusting the thickness of each layer, and the layered structure allows for emission from otherwise incompatible lanthanide pairs. This novel multi-layered approach enables high sensitivity over a broad temperature range. The highest relative sensitivity was achieved for a dual layered YVO_4_: Eu^3+^/YVO_4_: Dy^3+^ sample, exhibiting a maximum sensitivity of 3.6% K^−1^ at 640 K. The films were successfully deposited on all tested substrates (silicon, iron, aluminum, glass, quartz, and steel), and can be applied homogenously to most surfaces without the use of binders. The films are unaffected by water, enabling non-contact temperature sensing in water, where IR thermometers are not an option.

## Introduction

Luminescent temperature sensors are gaining attention as remote real-time temperature sensors due to their fast response, high spatial resolution and sensitivity, and low perturbation of the sample temperature during measurements. This enables them to be used for monitoring of moving parts, such as a working engine and *in vivo* temperature sensing of living organisms at the micro- and nanoscale^1–5^. As the signal from a luminescent probe is optical, it can pass through robust silica fibers, making them attractive for use in harsh conditions where the combination of electrical wiring and batteries is not a realistic option, e.g. in boreholes where temperatures can reach above 600 K^6,7^. The advantages of luminescent temperature sensors over other non-contact temperature sensors, such as IR-thermometers, are mainly their fast response, high spatial resolution and sensitivity, self-referencing, and that they do not rely on the emissivity of the material where the temperature is to be measured, as previously reviewed^8–10^.

Optical thermometry using luminescent materials can either be performed by measuring the changes in the lifetime of the excited state of the phosphor, or by measuring the difference in fluorescence intensity ratio (FIR) between two excited states that can either be thermally coupled (TCS)^3,11^, or non-thermally coupled (NTCS)^1,12–15^. Measurements based on the lifetime of temperature sensitive excited states requires sophisticated read out, time-consuming data fitting, and becomes less reliable if the lifetimes are not single-exponential. FIR measurements can be performed with simple read-out at significantly reduced cost. This signal is also intrinsically referenced, as variations in emission intensity, indicator concentration, geometry, source intensity, and light field are cancelled out when the relative emission of two different states are considered^9^. Probes based on the FIR methodology tend to either exhibit a high temperature sensitivity over a narrow operating range or poor sensitivity over a broad range due to how these parameters are linked. As luminescence eventually quenches with temperature, most materials do not exhibit sufficient emission intensity to be useful above 500 K, severely limiting the usefulness of FIR based probes for high-temperature applications. In this study, we demonstrate how it is possible to achieve high sensitivity over large operating ranges by employing multilayered thin films produced by the atomic layer deposition (ALD) technique.

**Figure 1:**
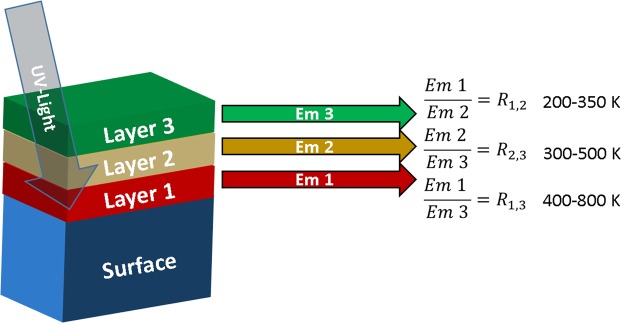
Concept of a thin-film material used for temperature detection over a wide temperature range. In this hypothetical material *R*_1,2_ is the FIR from a transition in layer 1 and layer 2, which is sensitive in the 200–350 K region, while *R*_2,3_ and *R*_1,3_ are sensitive in the 300–500 K and 400–800 K, respectively, thus yielding a highly sensitive temperature probe over the entire 200–800 K range.

A layered thin film can contain different luminescent ions in separate layers, resulting in numerous intensity ratios, each with an independent temperature sensitivity, similar to how e.g. YAG: Cr^3+^, Nd^3+^ exhibits three different kinds of FIRs in the same material^1^. By selecting luminescent ions whose emission quenches in different temperature ranges, it is possible to achieve high temperature sensitivity over a broad temperature range. Figure 1 shows a schematic of how a hypothetical material with several luminescent layers could work. The thickness of the layers needs to be carefully controlled in order to ensure that sufficient UV light is absorbed in each layer. The ALD technique exhibits excellent thickness control, and is thus particularly well suited for the fabrication of these materials.

While there may be TCS within each individual layer, there will be several NTCS that may be used for temperature sensing when considering the FIRs arising from the emission of the different layers. The emission ratio of NTCS is affected by several factors, such as particle size^12,16^, shape^17^, crystalline phase^18,19^, doping concentration^20^, and surrounding medium^21^. In layered samples, the luminescence is also affected by the thickness of the layers, and the order of the layers. As the thermal sensitivity depends on the luminescence intensity, there are consequently a wide range of parameters that can be used to further optimize the sensitivity for a specific temperature range.

In this study, we start by investigating how the emission in various single and dual layered YVO_4_: Ln^3+^ (Ln = Nd, Sm, Eu, Dy, Ho, Er, Tm, Yb) thin films deposited by ALD is affected by temperature in the 300–850 K range. We then demonstrate how ALD can be used to make layered films that contain different luminescent ions and thus provide ratiometric temperature sensitive emission over a broad temperature range. The films can be deposited on a probe and attached to a surface, or alternatively deposited directly on the surface where the temperature is to be determined.

YVO_4_ is a well-known host for lanthanides due to the strong absorption of (VO_4_)^3−^, fairly low phonon energies, high transparency in the visible range, non-hygroscopicity, and a low symmetry Y^3+^ site that can be substituted with lanthanides without distorting the structure^22–24^. As the films are only 100–150 nm thick, it is important that they are made of a highly absorbing material, and the films need to exhibit intense luminescence. Highly crystalline YVO_4_ is thus considered an optimal host. With YVO_4_ as host material, either temperature sensitive emission or excited state lifetimes have been reported for Eu^3+^ ^25^, Dy^3+^ ^10,26^, Nd^3+^ ^27–30^, and co-doped Er^3+^/Yb^3+^ ^31,32^, or Er^3+^/Ho^3+^ ^33^. Previously, we determined that crystalline growth of YVO_4_ can be achieved by using a 1-to-1 pulse ratio of Y(thd)_3_/O_3_ and VO(thd)_2_/O_3_ at 300 °C. The crystallinity, and as a consequence the luminescence intensity, can be further increased through post-deposition annealing^34^. Hansen *et al*. have previously reported on the growth of most of the lanthanide oxides from Ln(thd)_3_^35^, enabling the doping of lanthanides by substituting some of the Y(thd)_3_ pulses with other Ln(thd)_3_ pulses.

## Methods

The thin films investigated in this study were deposited with an F-120 research-type ALD-reactor (ASM Microchemistry Ltd) at 300 °C at a reactor pressure of ~3.6 mbar. The β-diketonate chelates Ln(thd)_3_ (Ln = Nd, Sm, Eu, Dy, Ho, Er, Tm, Yb, thd = 2,2,6,6-tetramethyl-3,5-heptanedione) were used as the lanthanide precursors. Y(thd)_3_, VO(thd)_2_ and O_3_ were used as the yttrium, vanadium and oxygen precursor, respectively. All information pertaining to the precursors is presented in Table 1. The concentration of the lanthanide doping relates to the amount of RE(thd)_3_ that is being pulsed. The actual concentrations are not determined in this study and will be termed as pulse% RE(thd)_3_, which will be abbreviated p% RE from here on. Previously, we have determined that the relation between p% and mol% is approximately 1:2 at these pulsing levels and a deposition temperature of 300 °C^34^, e.g. YVO_4_: 1 p% Dy^3+^ is expected to result in a Dy_0.02_Y_0.98_VO_4_ stoichiometry. Nitrogen was used as both carrier and purge gas and was supplied from gas cylinders (Praxair, 99.999%), run through a Mykrolis purifier, and maintained at a 300 cm^3^ min^−1^ primary flow rate. All depositions were preceded by an *in situ* 10 min ozone cleaning consisting of 100 cycles of 1 s O_3_ pulse and 5 s N_2_ purge at the deposition temperature in order to remove any organic remains while letting the reactor stabilize its temperature.

**Table 1:** Information on precursors used in this study.

Precursor	Producer	Purity	Sublimation temperature used [°C]	Pulse% cation/anion
VO(thd)_2_	In-house^55^		130	50
Y(thd)_3_	In-house^56^		130	48–49
Nd(thd)_3_	Strem Chemicals	>98 + % REO	165	1
Sm(thd)_3_	Strem Chemicals	>98 + % REO	145	1
Eu(thd)_3_	Strem Chemicals	>98 + % REO	145	2
Dy(thd)_3_	Strem Chemicals	>98 + % REO	130	1
Ho(thd)_3_	Strem Chemicals	>98 + % REO	130	1
Er(thd)_3_	Strem Chemicals	>98 + % REO	135	1
Tm(thd)_3_	Strem Chemicals	>98 + % REO	130	1
Yb(thd)_3_	Volatec	Not provided	130	1
O_3_	In USA ozone generator (AC-2025)	>99.9% O_2_		100

Pulse durations were 3/3/3/3/3/3/3/3 s for all the RE(thd)_3_/purge/O_3_/purge/VO(thd)_2_/purge/O_3_/purge cycles. These are longer pulse and purge times than what would be required to achieve saturating conditions for all precursors^35^, but optimizing these parameters was not the focus of this study and it was thus kept constant for all precursors. Si (100) and SiO_2_ were used as substrates for the depositions. The thickness of the native oxide layer on the silicon substrates ranged from 3–20 nm and was measured by spectroscopic ellipsometry prior to each deposition.

The deposited films have a thickness between 100–145 nm, which previously was determined to be sufficient in order for (VO_4_)^3−^ to absorb >90% of <300 nm light^34^. The samples are crystalline and luminescent as deposited, however, both these properties can be improved through post-deposition annealing above 700 °C. For each deposition, a sample was annealed post deposition in air at 1000 °C for 10 h. The graphs, images and figures in this study refers to annealed samples unless states otherwise.

The film thickness was determined with a J. A. Woollam alpha-SE ellipsometer in the 380–890 nm range. The Cauchy-model was used to parameterize the ellipsometry experimental data. Photoluminescence (PL) measurements in the visible range were performed with a 280 nm 1 mW diode and an OceanOptics USB4000 spectrometer for the high-resolution measurements shown in the supplementary section, while an USB2000+ spectrometer with high sensitivity was used for all the spectra collected above 300 K presented in the paper. An OceanOptics NIR-Quest spectrometer was used for recording emission spectra in the 900–1700 nm NIR. The detectors were not calibrated with respect to each detectors wavelength dependent response, and while this affects the absolute FIR values, it does not affect the relative change in the FIR and consequently not the reported relative sensitivity.

The temperature sensitivity measurements were conducted by positioning the samples vertically inside a tube furnace and threading a quartz rod through a hole in the insulation. This setup allows photons to travel from the excitation source through a split fiber and then through the quartz rod aimed at the sample inside the furnace. The emitted light from the sample is passed back through the rod and the split fiber, before being collected at the spectrometer. A thermocouple was used to monitor the temperature inside the furnace. The resolution of the controller for the thermocouple was 1 K. The samples were heated to 700–875 K at a ramp rate of 2 K min^−1^, and then kept at the max temperature for 10 min, before being cooled down by turning off the heater (ca. −0.5 K min^−1^ on average).

The crystallinity of the samples was determined with a Bruker D8 Discovery X-ray diffractometer, using CuK_α1_ radiation and a Ge(111) monochromator. UV-Vis measurements were conducted on films deposited on fused silica in the range 200–900 nm with a Shimadzu UV-3600 instrument and an integrating sphere. A Hitachi SU8230 field emission scanning electron microscope (FE-SEM) was used to study the surface morphology of some of the samples.

## Results and Discussion

**Figure 2:**
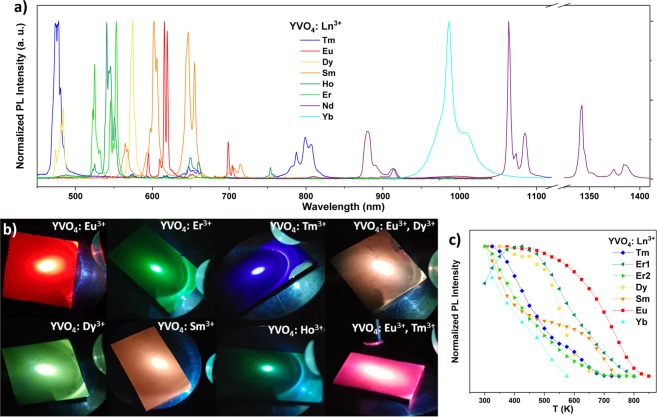
(**a**) Emission spectra of various lanthanides in YVO_4_ thin films in the range 450–1410 nm. (**b**) Photographs of a selection of the annealed thin-films while being excited by a 280 nm diode. (**c**) The temperature dependence of the total measured emission intensity, resulting from emission from the most emissive state, for various lanthanides in YVO_4_. Er1 and Er2 refer to emission from ^2^H_11/2_ and ^4^S_3/2_, respectively.

As only YVO_4_: Yb^3+^ had been previously deposited by ALD^34^, the first step to making multilayered films was to deposit YVO_4_ films doped with different lanthanides by ALD and to determine which of them are most promising for temperature sensing in multilayered films. The normalized emission spectra of all the deposited YVO_4_: Ln^3+^ (Ln^3+^ = Eu^3+^, Dy^3+^, Tm^3+^, Ho^3+^, Er^3+^, Sm^3+^, Nd^3+^, Yb^3+^) thin films after annealing are presented in Fig. 2a. Films with YVO_4_: 1 p% Tb^3+^ and YVO_4_: 1 p% Pr^3+^ were also prepared, but exhibited no detectable luminescence, which is to be expected since these ions easily oxidize to Tb^4+^ and Pr^4+^. The as-deposited samples are crystalline with a highly preferential orientation along the growth direction (shown for two samples in Fig. S1) and exhibit luminescence. Their emission intensity is further improved by post-deposition annealing, which we previously have shown for YVO_4_: Yb^3+^ is due to increased crystallinity^34,36^. XRD data of the samples whose temperature sensing properties were determined in the present study is provided in Fig. S2. Individual emission spectra with peak labels for each ion is provided in Figs S3–10, which for YVO_4_: Er^3+^ also includes emission in the 1450–1650 nm range. Transmission measurements of a selection of the samples are shown in Fig. S11, while photographs of samples exhibiting strong visible emission, including two dual layered samples, are presented in Fig. 2b.

Among the lanthanides whose emission is depicted in Fig. 2a, Eu^3+^, Dy^3+^, Er^3+^, Ho^3+^ and Nd^3+^ exhibit significant emission from TCS, enabling them to be used in ratiometric measurements. Kalinichev *et al*. previously performed a dedicated study on the temperature sensitivity of YVO_4_: Nd^3+^ nanoparticles, where they investigated the intensity ratio between the ^4^F_3/2_–^4^I_9/2_ and ^4^F_5/2_ + ^2^H_9/2_–^4^I_9/2_ transitions, finding sensitivities ranging from 9% K^−1^ at 123 K, to 0.18% K^−1^ at 873 K^27^. The sensitivity in the lower temperature range is remarkable, however, the rapidly quenched luminescence means that the sensitivity is poor above 300 K. YVO_4_: Nd^3+^ was consequently not investigated in the present study. Additionally, the YVO_4_: Ho^3+^ sample was omitted from further investigations due to its fairly weak emission compared to other samples emitting in the visible range, and previously reported sensitivities for Ho^3+^ in ceramic glass are low (maximum of 0.1% K^−1^)^37^.

In order to understand how the emission of the various lanthanides changes with temperature in YVO_4_ thin films, a preliminary investigation was done where the total emission from the most emissive state of each sample was monitored in the 300–875 K range (Fig. 2c). The signal measured in this way is not referenced, so the results is merely an indication of the temperature range where the various lanthanides can be used as temperature sensors. YVO_4_: Eu^3+^ emission has the highest quenching temperature, and should be included if the goal is to develop a sensor with high sensitivity above 600 K. In this study, we have first investigated single layer materials of two different lanthanides with TCS, before investigating two combinations of lanthanides suitable in the 473–773 K range.

### YVO_4_: Er^3+^

The visible range of the YVO_4_: Er^3+^ emission spectrum is dominated by two peaks resulting from the ^2^H_11/2_–^4^I_15/2_ and the ^4^S_3/2_–^4^I_15/2_ transitions (for high-resolution spectrum see Fig. S6). The small energy gap separating these TCS causes significant overlap in their emission in most host structures, causing large detection errors^38–40^. Nevertheless, Er^3+^ has been extensively studied for use as a temperature probe due to these TCS^41,42^, though exclusively when co-doped with Yb^3+^ or Nd^3+^ as an upconversion phosphor, primarily for *in vivo* applications. Upconversion phosphors require high excitation power in order to perform, while regular phosphors can be easily excited by an inexpensive 1 mW UV-diode.

**Figure 3:**
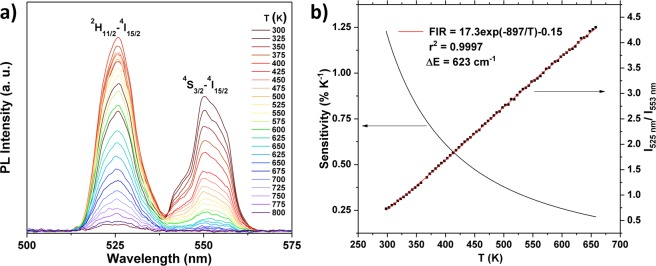
(**a**) Emission spectra of YVO_4_: 1 p% Er^3+^ in the temperature range 300–800 K. (**b**) The temperature dependence of the FIR between the emission peaks at 525 nm (^2^H_11/2_–^4^I_15/2_) and 553 nm (^4^S_3/2_–^4^I_15/2_). The red line shows the best fit for the experimental data (black squares) to the equation: FIR = C exp(Δ*E* *k*_B_^−1^ *T*^−1^). The black solid line shows the corresponding relative temperature sensitivity. An Arrhenius-plot representation of the data is available in Fig. S12.

In the YVO_4_: 1 p% Er^3+^ thin film presented in this study, the overlap between the emission from the ^2^H_11/2_–^4^I_15/2_ and the ^4^S_3/2_–^4^I_15/2_ transitions appear to be negligible when using detectors with high resolution. Figure 3a shows the thermal dependence of the emission from an YVO_4_: 1 p% Er^3+^ thin film, using a detector with lower resolution, but higher sensitivity. The overlap is still small enough to not cause significant detection errors. The emission intensity, *I*, for each transition is proportional to the population of atoms in a given excited state at temperature *T*^3^:

1 $${\boldsymbol{I}}\propto {\boldsymbol{g}}{\boldsymbol{A}}{\boldsymbol{h}}{\boldsymbol{v}}\,\exp (\,-\frac{{\boldsymbol{E}}}{{{\boldsymbol{k}}}_{{\boldsymbol{B}}}{\boldsymbol{T}}}),$$

where *g* is the degeneracy of the state, *A* is the spontaneous emission rate, *h* is the Planck constant, *v* is the frequency, and *E* is the energy of the level. The FIR between two transitions assigned to the same phosphor, the lower level *I*_1_ and upper level *I*_2_, is taken as a measure of absolute temperature due to:

2 $$\frac{{{\boldsymbol{I}}}_{2}}{{{\boldsymbol{I}}}_{1}}=\frac{{{\boldsymbol{g}}}_{2}{{\boldsymbol{A}}}_{2}{\boldsymbol{h}}{{\boldsymbol{v}}}_{2}}{{{\boldsymbol{g}}}_{1}{{\boldsymbol{A}}}_{1}{\boldsymbol{h}}{{\boldsymbol{v}}}_{1}}\exp (\,-\frac{{\rm{\Delta }}{{\boldsymbol{E}}}_{21}}{{{\boldsymbol{k}}}_{{\boldsymbol{B}}}{\boldsymbol{T}}})={\boldsymbol{C}}\,\exp (\,-\frac{{\rm{\Delta }}{{\boldsymbol{E}}}_{21}}{{{\boldsymbol{k}}}_{{\boldsymbol{B}}}{\boldsymbol{T}}}),$$

where *C* is the temperature-independent scaling constant, and Δ*E*_21_ is the energy gap between the two TCS. In Er^3+^, the ^4^S_3/2_ state is considered the lower level, while the ^2^H_11/2_ state is considered the upper level. Δ*E* between two TCS can be obtained by fitting experimental data of the FIR to eq. 2, as shown for YVO_4_: Er^3+^ in Fig. 3b. A good fit was achieved for the 300–660 K range (*r*^2^ = 0.9997), providing a fitted value for Δ*E* of 623 cm^−1^, which is in good agreement with the emission spectra (ca. 650 cm^−1^). The relative thermal sensitivity, *S*, of the probe, defined as relative change in the FIR with temperature^43^:

3 $${\boldsymbol{S}}=\frac{1}{{\bf{F}}{\bf{I}}{\bf{R}}}\frac{\Delta {\bf{F}}{\bf{I}}{\bf{R}}}{{\rm{\Delta }}{\boldsymbol{T}}}\cdot 100 \% ,$$

and is also shown in Fig. 3b. Note that the first derivative of the FIR gives a measure of the absolute sensitivity, and depends on the absolute FIR value, which as mentioned previously may depend on power density, particle size^12,16^, shape^17^, crystalline phase^18,19^, doping concentration^20^, surrounding medium^21^, and in layered samples also the thickness of the layers and the order of the layers. Therefore, while the absolute sensitivity is relevant for the practical operation, it is not a meaningful parameter for comparison between probes, and thus only the relative sensitivities will be presented in this study. For the investigated temperature range, the *S* of this sample spans from 1.23% K^−1^ at 300 K to 0.21% K^−1^ at 660 K, making it reasonable for measuring temperature changes in e.g. water. The FIR between 295–370 K, and a picture of the experimental setup of YVO_4_: 1 p% Er film submerged in water, is given in Fig. S13. Due to the non-hygroscopicity of YVO_4_, the Er^3+^ emission is unaffected by water, which will be demonstrated for YVO_4_: Eu^3+^ in the next section, and the resulting FIR is thus similar to that of Fig. 3b in the relevant temperature range.

### YVO_4_: Eu^3+^

Eu^3+^ emission has previously been investigated for use as ratiometric temperature sensors in several hosts^44–51^, including YVO_4_^25^. Previous studies have primarily investigated the thermal dependence of FIR between the emission from the ^5^D_1_ and ^5^D_0_ excited states. The study on YVO_4_: Eu^3+^ investigated ratios between the emissions from two stark sublevels of the ^7^F_2_ excited state the 293–333 K range, with *S* being in the order of 0.1% K^−1^. As the emission from these two levels overlaps significantly (Fig. S3), in addition to the relatively poor reported sensitivity, it is evident that the FIR based on emission from these states is not well suited for use as a temperature sensor. In this study, we investigate the FIR between the emission of the ^5^D_0_–^7^F_4_ transition and the ^5^D_1_–^7^F_1_ transition.

**Figure 4:**
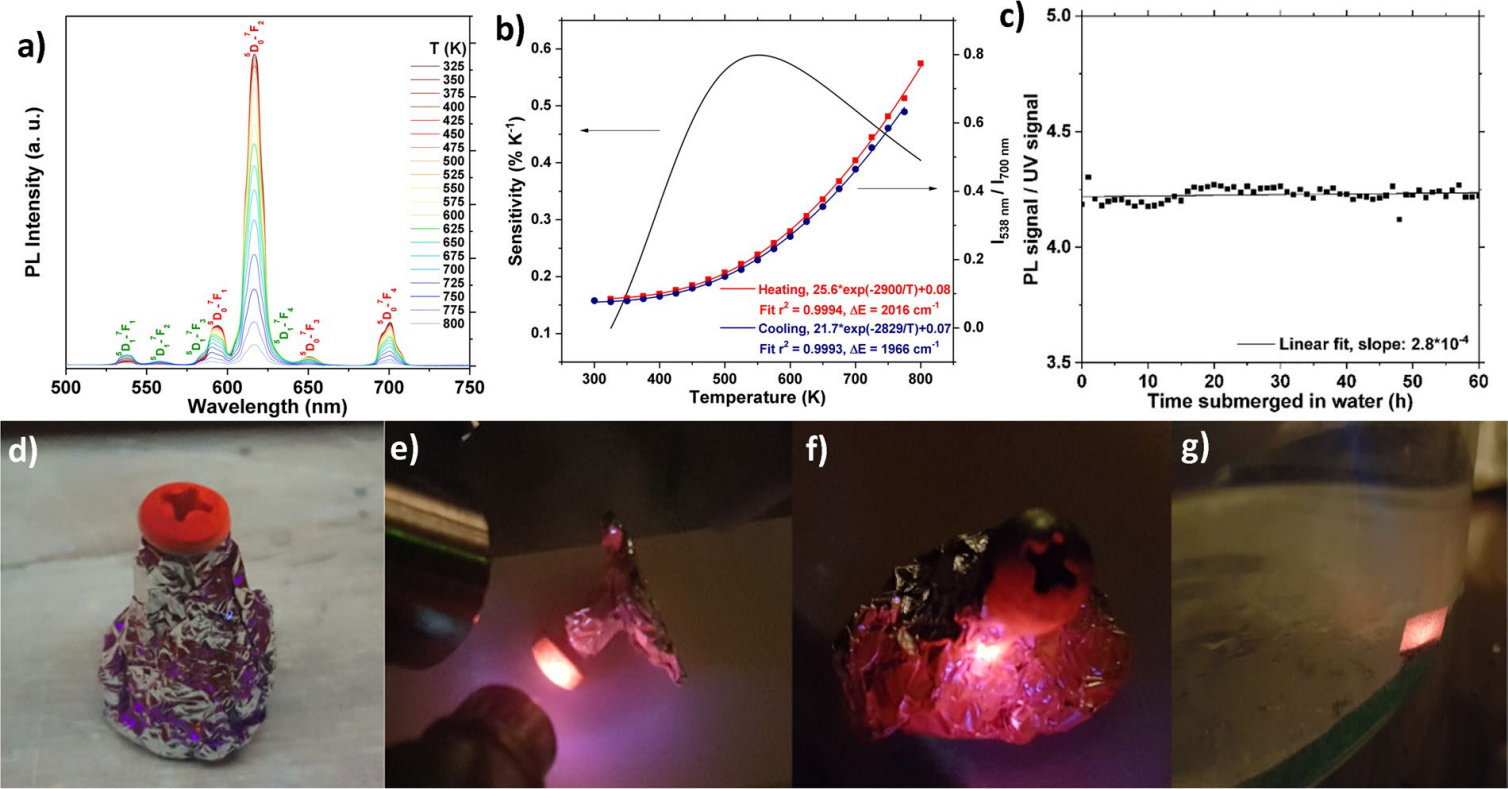
(**a**) Emission spectra of YVO_4_: 2 p% Eu^3+^ in the 325–800 K range. Transitions from the ^5^D_1_ excited states are marked in green, while transitions from the ^5^D_0_ excited states are marked in red. (**b**) The thermal dependence of the FIR between the emission at 538 nm (^5^D_1_–^7^F_1_) and 700 nm (^5^D_0_–^7^F_4_). The red line shows the best fit for the experimental data (black squares) to the equation: FIR = C exp(Δ*E* *k*_B_^−1^ *T*^−1^). The black solid line shows the corresponding relative temperature sensitivity. (**c**) Total Eu^3+^ emission divided by total reflected UV signal from the diode of the sample submerged in water for 60 hours. (**d**) Photograph of a screw and Al-foil coated by as-deposited YVO_4_: 2 p% Eu^3+^ while being illuminated by 254 nm light at 293 K. (**e**) A different screw illuminated by a 280 nm diode while being heated (ca. 350 K) by a heat-gun (note that the diode emits some blue light that affects the color). (**f**) Al-foil used to keep the screw in place during deposition being illuminated by UV-light at 293 K. (**g**) An annealed YVO_4_: 2 p% Eu^3+^ sample excited by 280 nm UV-light while being submerged in water (293 K).

The thermal dependence of the emission spectrum of YVO_4_: 2 p% Eu^3+^ is presented in Fig. 4a. While the ^5^D_0_–^7^F_2_ emission is by far the strongest emission, it overlaps with both the emission from the ^5^D_0_–^7^F_1_ and ^5^D_1_–^7^F_4_ transitions, making accurate background subtraction challenging. Good agreement between heating and cooling measurements was obtained by instead using the intensity of the ^5^D_0_–^7^F_4_ transition. Figure 4b shows the experimental data fitted to eq. 2 (*r*^2^ = 0.9994). The average experimental value for Δ*E* was determined to be 1991 cm^−1^, which is in good agreement with previous studies^45^. A lower Δ*E* (1872 cm^−1^), which is closer to the experimental value suggested by the PL spectra (1735 cm^−1^), is obtained by extracting Δ*E* from an Arrhenius-plot representation of the data, as shown in Fig. S14.

Figure 4b also shows the thermal dependence of *S*, which reaches a maximum of 0.59% K^−1^ at 550 K. While *S* is fairly low, it is still higher than e.g. YVO_4_: Er^3+^ in the 450–800 K range, making YVO_4_: Eu^3+^ a reasonable alternative for measurements in this temperature range. Discrepancies between heating and cooling are attributed to thermal mass in the system, and that the thermocouple used to measure the temperature was positioned 1–2 cm from the sample.

As YVO_4_ is non-hygroscopic material, it will not absorb water, and submerging the films in water should thus not affect their luminescence. This was verified by submerging an YVO_4_: 2 p% Eu^3+^ sample in water for 60 hours while recording the PL emission. The total emission of the sample depends proportionally on the power of the excitation source, which fluctuates significantly over the course of such a long experiment. Changes in the background, in the atmosphere, or with the equipment is only expected to yield minor variations. Instability of the excitation source can be accounted for by recording the reflected UV signal (Fig. S15). Figure 4c shows how the total emission divided by the total reflected UV emission changes over the course of 60 hours while the sample is submerged in water. Fitting the data to a linear function shows that there is an insignificant increase in emission, and thus implies that the samples are completely unaffected by water. This enables monitoring of the temperature of transparent liquids inside e.g. sealed ampoules, Schlenk lines, or hydrothermal pressure vessels, provided there is an optical window that the signal can pass through. Note that underwater temperature sensing is not possible with IR thermometers due to the IR absorption of water, and that YVO_4_ based temperature sensors thus provide a new solution to measuring in this condition.

Figure 4d,e shows two different screws, propped up with Al-foil during deposition, and coated with an as-deposited YVO_4_: 2 p% Eu^3+^ ALD-thin film, while being exposed to two different sources of UV light. Figure 4f shows the luminescence from the Al-foil used to keep the screws in place during the deposition. The images demonstrate that it is possible to deposit luminescent YVO_4_: Ln^3+^ films on other surfaces than Si, glass or quartz. Heat conducting screws, clips, foils and similar items can also act as probes that can be attached to the desired area, in cases where direct deposition is inconvenient or impossible. Figure 4g shows the emission from an YVO_4_: 2 p% Eu^3+^ sample submerged in water. Note that it is also possible to cap ALD-films with a protective layer of Al_2_O_3_, or other transparent materials, in order to protect the coating from various chemical and physical environments.

### Layered YVO_4_: Eu^3+^/YVO_4_: Dy^3+^

In addition to using two TCS on the same lanthanide, it is also possible to use the intensity ratio of two different lanthanides resulting from NTCS. In layered YVO_4_: Eu^3+^/YVO_4_: Dy^3+^, this results in at least six FIRs that can be extracted from the same signal. Both Eu^3+^ and Dy^3+^ exhibit two TCS, resulting in an additional four NTCS that can be used for temperature determination. A previous study on a similar system was recently performed by Wang *et al*. on SrWO_4_: Eu^3+^, Dy^3+^ co-doped phosphor, yielding an impressive maximum *S*, *S*_max,_ of 1.71% K^−1^ at 335 K when using the FIR between the ^4^F_9/2_–^6^H_13/2_ emission of Dy^3+^ and the ^5^D_0_–^7^F_2_ emission of Eu^3+^ ^13^. It is thus evident that this kind of approach can be fruitful for developing optical temperature sensors with high *S* over a broad temperature range. The TCS in YVO_4_: Dy^3+^ was recently investigated, demonstrating a *S*_max_ of 1.8% K^−1^ at 298 K, with a monotonic decrease to ca. 0.4% K^−1^ at 673 K^26^. Due to non-linear background intensity from the diode used in the present study in the 450 nm range, the ^4^I_15/2_–^6^F_15/2_ emission of Dy^3+^ in the 455 nm range was challenging to determine accurately. The FIR from the TCS and corresponding *S* for an YVO_4_: 1 p% Dy^3+^ sample are nevertheless available in Fig. S16. Values for *S* are similar those from Kolesnikov *et al*. above 400 K, but significantly larger below. Other FIRs based on the ^4^I_15/2_–^6^F_15/2_ emission of Dy^3+^ have thus been omitted from this study, but it is important to note that these FIRs are still available to use in practical operation with a suitable excitation source.

**Figure 5:**
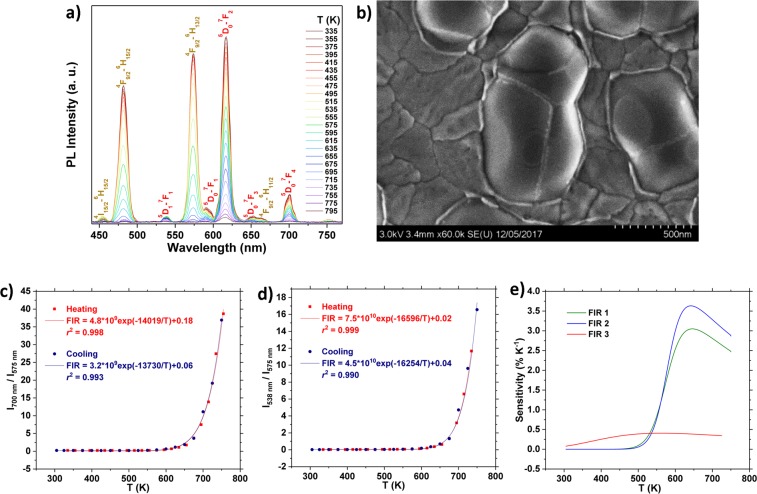
(**a**) Emission spectra of a layered 78 nm YVO_4_: 2 p% Eu^3+^/39 nm YVO_4_: 1 p% Dy^3+^ thin film in the temperature range 330–795 K. Dy^3+^ transitions marked in yellow, while Eu^3+^ transitions are marked in red. (**b**) FE-SEM image of layered YVO_4_: Eu^3+^/YVO_4_: Dy^3+^ annealed at 1000 °C. (**c**) Thermal dependence of the FIR of the 575 nm (^4^F_9/2_–^6^H_13/2_) emission of Dy^3+^ and 700 nm (^5^D_0_–^7^F_4_) emission of Eu^3+^ (FIR 1). (**d**) Thermal dependence of the FIR between the 575 nm (^4^F_9/2_–^6^H_13/2_) emission of Dy^3+^ and the 538 nm (^5^D_1_–^7^F_1_) emission of Eu^3+^ (FIR 2). (**e**) The thermal dependence of *S* for the different FIRs in an YVO_4_: 2 p% Eu^3+^/YVO_4_: 1 p% Dy^3+^ thin film. FIR 1: 575 nm emission of Dy^3+^ and 700 nm emission of Eu^3+^ (green), FIR 2: 575 nm emission of Dy^3+^ and 538 nm emission of Eu^3+^ (blue), FIR 3: 700 nm and 538 nm emission of Eu^3+^ (red).

In the films investigated in the present study, the ^4^F_9/2_–^6^H_13/2_ Dy^3+^ emission quenches quite abruptly in the 500–750 K range, while the Eu^3+^ emission quenches at a slower rate at these temperatures (Fig. 2c), resulting in high *S* in this range. Figure 5a shows how the emission of an annealed sample with 39 nm YVO_4_: 1 p% Dy^3+^ deposited on top of 78 nm YVO_4_: 2 p% Eu^3+^ changes with temperature. A FE-SEM image of the surface of this sample is shown in Fig. 5b.

Note that while it has been demonstrated that the FIR methodology can still be used even when there is no thermal coupling between the involved states^14,52^, the number of possible excitation and de-excitation processes that affect the emission in these systems can be large. Finding an analytical solution to the rate equation system that describes the whole phenomena is consequently non-trivial^12^. However, in order to determine *S* for the system, it is only necessary that the data can be fitted by a function that adequately captures the changes in the experimental data. In previous studies on systems with NTCS, it was observed that all the FIRs presented an approximate exponential growth with the measured temperatures, similar to that of TCS. This suggests that the same equations can be used to fit the data, despite the physical interpretation of each parameter being unclear^12,52^. The FIR of the 575 nm (^4^F_9/2_–^6^H_13/2_) emission of Dy^3+^ and 700 nm (^5^D_0_–^7^F_4_) emission of Eu^3+^ (FIR 1) is presented in Fig. 5c. The best fit to eq. 1 is reasonable (*r*^2^ = 0.998), and seems to capture the changes in the FIR well in the temperature sensitive 500–750 K range.

The ratio between the ^4^F_9/2_–^6^H_13/2_ emission of Dy^3+^ and the ^5^D_1_–^7^F_1_ transition of Eu^3+^ (FIR 2) is presented in Fig. 5d, and this data could also be fitted well to eq. 1 (*r*^2^ = 0.999). The FIR that was used in YVO_4_: Eu^3+^, i.e. the ratio between the 538 nm and 700 nm emission of Eu^3+^, is also present in this sample (FIR 3). The thermal dependence of this FIR is given in Fig. S17, and results in a slightly lower *S* compared to a sample without any Dy^3+^.

The thermal dependence of *S* of the three FIRs in the layered YVO_4_: 2 p% Eu^3+^/YVO_4_: 1 p% Dy^3+^ thin film is presented in Fig. 5e. The addition of an YVO_4_: Dy^3+^ layer on top of YVO_4_: Eu^3+^ enhances *S* significantly in the 500–750 K range, with a maximum of 3.6% K^−1^ at 640 K. The FIR resulting from TCS on Dy^3+^ is not included in Fig. 5e, but exhibits *S* of 0.9–1.8% K^−1^ below 400 K^26^, enabling high *S* over the entire 300–800 K range.

A list of phosphor materials that can be used for temperature detection above 473 K, and their *S*_max_ and operating range is presented in Table 2. It is challenging to make a useful comparison between various systems based on *S*_max_ due to the differences in operating range. While the listed systems still exhibit luminescence above 473 K, their *S*_max_ may be at a much lower temperature, and the sensitivity in the 473–750 K range may be significantly lower than the value listed. E.g. the *S*_max_ observed for the YVO_4_: Er^3+^ film presented in this study was 1.2% K^−1^ at 300 K, while above 473 K, *S* is less than 0.5% K^−1^ and gradually decreasing, resulting in an average *S* of only 0.30% K^−1^ in the 473–673 K range. On the other hand, the dual-layer YVO_4_: Eu^3+^/YVO_4_: Dy^3+^ thin film has an average *S* of 2.2% K^−1^ over the 475–750 K range (2.4% in the 500–750 K range). An average sensitivity over a certain operating range would be more sensible to compare, though such numbers have not been compared in previous studies, as such comparisons only make sense if the systems are compared for use in a specific application requiring a certain operating range. A recent study on the Pr^3+^: Y_2_Ti_2_O_7_ system, using the FIR of host trap emission and the ^1^D_2_–^3^H_4_ emission of Pr^3+^, demonstrated an impressive *S*_max_ of 5.25% K^−1^ at 289 K in the operating range 289–573 K^53^. However, above 473 K it is less than 1.5% K^−1^ and decreasing, clearly making it less suitable than the dual layer YVO_4_: Eu^3+^/YVO_4_: Dy^3+^ thin film in the 500–750 K range. To the authors’ knowledge, there are no previously investigated materials that exhibit higher average *S* than that of the dual layer YVO_4_: Eu^3+^/YVO_4_: Dy^3+^ presented in this study for FIR measurements in the 500–750 K temperature range, making this currently the most attractive material for non-contact temperature sensing in this range.

**Table 2:** The maximum relative sensitivities, *S*_max_, and operating temperature range (within limits of measurement) for selected phosphors that can operate *above* 473 K. *Derived from FIR data.

System	Transitions	*S*_max_ [% K^−1^]	Range [K]	Source
YVO_4_: Er^3+^	^2^H_11/2_ – ^4^I_15/2_ / ^4^S_3/2_ – ^4^I_15/2_	1.2	300–675	This work
YVO_4_: Eu^3+^	^5^D_1_–^7^F_1_ / ^5^D_0_ – ^7^F_4_	0.6	300–800	This work
YVO_4_: Dy^3+^	^4^I_15/2_^– 6H^_15/2_ / ^4^F_9/2_ – ^6^H_13/2_	1.8	298–673	^26^
YVO_4_: Eu^3+^, Dy^3+^	Eu^3+ 5^D_0_ – ^7^F_4_ / Dy^3+ 4^F_9/2_ – ^6^H_13/2_	3.6	500–750	This work
YVO_4_: Eu^3+^, Dy^3+^	Eu^3+ 5^D_1_ – ^7^F_1_ / Dy^3+ 4^F_9/2_ – ^6^H_13/2_	3.0	500–750	This work
YVO_4_: Tm^3+^, Eu^3+^	Tm^3+ 1^G_4_ – ^3^H_6_ / Eu^3+ 5^D_1_ – ^7^F_1_	1.9	500–775	This work
Y_2_O_3_: Er^3+^ nanoparticles	^2^H_11/2_ – ^4^I_15/2_ / ^4^S_3/2_ –^4^I_15/2_	1.5	296–500	^12^
Tb^3+^/Pr^3+^:NaLu(WO_4_)_2_	^5^D_4_ – ^7^F_5_, ^1^D_2_ – ^3^H_4_	1.45	583–783	^53^
GC: Ho^3+^	^5^F_2,3_/^3^K_8_ – ^5^I_8_^5^F_1_ / ^5^G_6_ – ^5^I_8_	0.1	303–643	^37^
β-NaYF_4_: Nd^3+^	^4^F_7/2_ – ^4^I_9/2_ / ^4^F_3/2_ – ^4^I_9/2_	1.1	323–673	^57^
La_2_O_3_: Yb^3+^, Nd^3+^	^4^F_7/2_ – ^4^I_9/2_ / ^4^F_5/2_ – ^4^I_9/2_	1.4	300–1200	^4^
Dual phase GC: Cr^3+^, Tb^3+^	^2^E – ^4^A_2_ / ^5^D_4_ – ^7^F_5_	0.6*	300–573	^15^

### Layered YVO_4_: Tm^3+^/YVO_4_: Eu^3+^

**Figure 6:**
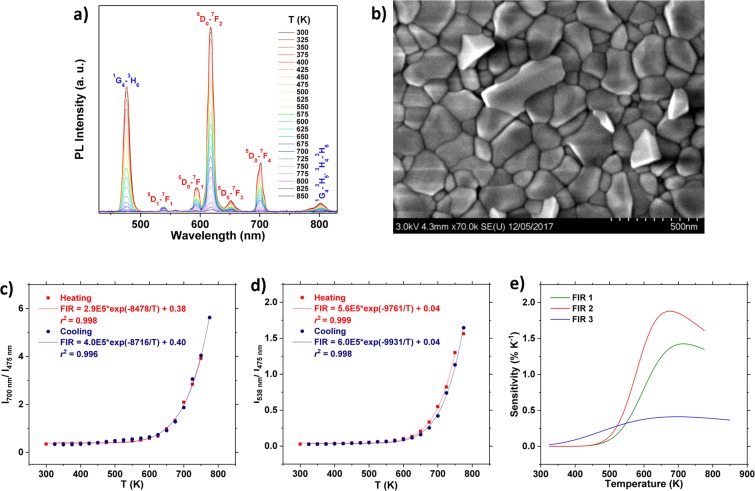
(**a**) Emission spectra of a layered 125 nm YVO_4_: 1 p% Tm^3+^/20 nm YVO_4_: 2 p% Eu^3+^ thin film in the 300–850 K range. (**b**) FE-SEM image of layered YVO_4_: Tm^3+^/YVO_4_: Eu^3+^ after annealing at 1000 °C. (**c**) FIR of the 475 nm (^1^G_4_–^3^H_6_) emission of Tm^3+^ and the 700 nm (^5^D_0_–^7^F_4_) emission of Eu^3+^ (FIR 1) best fit to the experimental data. (**d**) FIR of the 475 nm (^1^G_4_–^3^H_6_) emission of Tm^3+^ and the 538 nm (^5^D_1_–^7^F_1_) emission of Eu^3+^ (FIR 2). The data point at 775 K for the heating experiment was not included in the fit due to poor signal to noise ratio. (**e**) The thermal dependence of *S* for the different FIRs in the YVO_4_: Tm^3+^/YVO_4_: Eu^3+^ system.

YVO_4_: Tm^3+^/YVO_4_: Eu^3+^ is another dual layered material that seemed promising based on Fig. 2c. YVO_4_: Tm^3+^ quenches at a slower rate, but over a slightly broader temperature range compared to YVO_4_: Dy^3+^. The emission spectra in the 300–850 K temperature range of a dual layered thin film consisting of 20 nm YVO_4_: 2 p% Eu^3+^ deposited on top of 125 nm YVO_4_: 1 p% Tm^3+^ is presented in Fig. 6a, while a FE-SEM image of the surface of the sample is provided in Fig. 6b. The strong Eu^3+^ emission indicates that the large difference in thickness between the Eu^3+^ and Tm^3+^ layer is required for a balanced ratio of the emission intensity. It has previously been determined that there is resonant energy transfer from Tm^3+^ to Eu^3+^ ^54^, and thus the emission properties will be dominated by Eu^3+^ when mixed together. By making a layered structure, the energy transfer from Tm^3+^ to Eu^3+^ can be reduced, enabling intense emission from both lanthanides.

In the YVO_4_: Tm^3+^, Eu^3+^ system there are at least three FIRs suitable for temperature measurements. The FIR of the ^1^G_4_–^3^H_6_ transition of Tm^3+^ and ^5^D_0_–^7^F_4_ of Eu^3+^ is presented in Fig. 6c (FIR 1). It is evident that the FIR increases exponentially, and the best fit to eq. 2 is reasonable (*r*^2^ = 0.998), indicating that this is indeed a valid approach for this system as well. The FIR of the ^1^G_4_–^3^H_6_ transition of Tm^3+^ and ^5^D_1_–^7^F_1_ transition of Eu^3+^ is presented in Fig. 6d (FIR 2), while the FIR of the ^5^D_0_–^7^F_4_ and the ^5^D_1_–^7^F_1_ transition of Eu^3+^ in this sample (FIR 3) is provided in Fig. S18. Figure 6e shows a comparison between the thermal dependence of *S* of the three FIRs. FIR 2 exhibits a maximum *S* of 1.9% K^−1^ at 675 K, and displays a reasonable sensitivity over the 500–775 K range (average *S* = 1.35% K^−1^). *S* is lower compared to YVO_4_: Dy^3+^/YVO_4_: Eu^3+^, but the operating range is slightly broader.

## Conclusion

In this study, controlled deposition of luminescent YVO_4_: Ln^3+^ (Ln = Nd, Sm, Eu, Dy, Ho, Er, Tm, Yb) by ALD was achieved, both as single layers and as multilayers, and the thermal dependence of the photoluminescence in the 300–875 K range of the samples doped with Eu^3+^, Dy^3+^, Tm^3+^, Sm^3+^, Er^3+^ and Yb^3+^ was investigated. FIR measurements using TCS on Er^3+^ and Eu^3+^ demonstrated maximum sensitivities reaching 1.2% K^−1^ for YVO_4_: Er^3+^ at 300 K, and 0.6% K^−1^ for Eu^3+^ at 575 K. FIR measurements were also performed using excited states situated on different lanthanides in layered YVO_4_: Eu^3+^/YVO_4_: Dy^3+^ and YVO_4_: Tm^3+^/YVO_4_: Eu^3+^. The dual layered materials exhibit a *S*_max_ of 3.6% K^−1^ at 640 K, with *S* significantly improved in the whole 500–775 K range compared to the single layer YVO_4_: Eu^3+^ thin film, and also compared to any other previously investigated materials in this range. The dual layered films are particularly attractive for use in boreholes where the temperatures exceed 500 K. The films were successfully deposited on all tested surfaces (Si, Fe, Al, glass, quartz, and steel), indicating that they can be applied to most industrially relevant surfaces. It was also demonstrated that the luminescence of the films is unaffected by water, which enables monitoring of the temperature of transparent liquids inside e.g. sealed ampoules, Schlenk lines or hydrothermal pressure vessels. The study shows that it is possible to make highly customizable, chemically and physically robust coatings by ALD that can provide ratiometric temperature sensing over a broad temperature range and with high sensitivity.

## Supplementary information

Figure S1–18

## Data Availability

The experimental data is available upon request or can be accessed from the Zenodo repository.
