# Supplementary material for: Sensors for optical thermometry based on luminescence from layered YVO4: Ln3+ (Ln = Nd, Sm, Eu, Dy, Ho, Er, Tm, Yb) thin films made by atomic layer deposition
Source: Sci Rep. 2019 Jul 15;9:10247. doi: 10.1038/s41598-019-46694-8 (PMC6629663; doi:10.1038/s41598-019-46694-8)
Supplement: Supplementary file 1 — Figure S1–18 [file 41598_2019_46694_MOESM1_ESM.pdf]

Supplementary Material

Sensors for optical thermometry based on luminescence from layered YVO_4: Ln^{3+} (Ln = Nd, Sm, Eu, Dy, Ho, Er, Tm, Yb) thin films made by atomic layer deposition

Michael N. Getz, Ola Nilsen, and Per-Anders Hansen

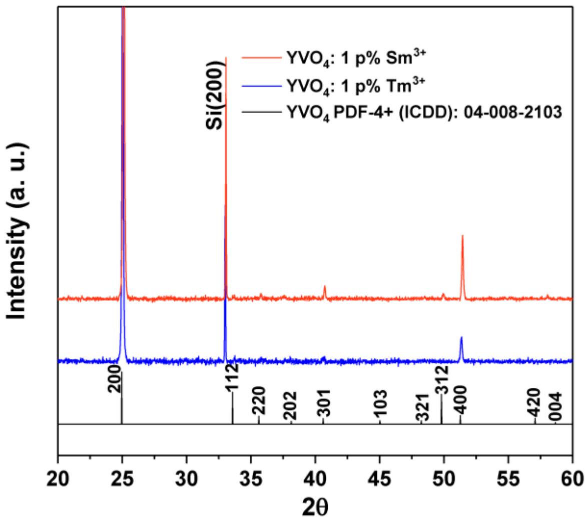

Figure S1 XRD patterns of as deposited YVO_4: 1 p% Sm^{3+} and YVO_4: 1 p% Tm^{3+}, compared with pattern of YVO_4.XRD patterns of YVO_4: 1 p% Er^{3+}, YVO_4: 2 p% Eu^{3+}, dual layered YVO_4: 2 p% Eu^{3+} / YVO_4: 1 p% Dy^{3+}, and dual layered YVO_4: 2 p% Eu^{3+} / YVO_4: 1 p% Tm^{3+} after annealing at 1000 ˚C for 1 hour, compared with the XRD pattern of pure YVO_4 powder.[58]

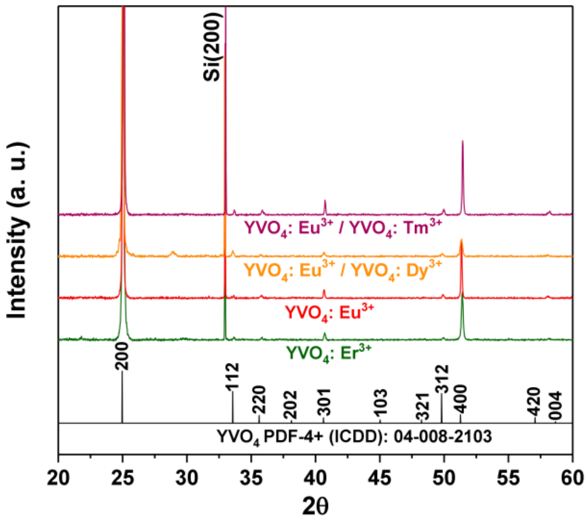

Figure S2 XRD patterns of YVO_4: 1 p% Er^{3+}, YVO_4: 2 p% Eu^{3+}, dual layered YVO_4: 2 p% Eu^{3+} / YVO_4: 1 p% Dy^{3+}, and dual layered YVO_4: 2 p% Eu^{3+} / YVO_4: 1 p% Tm^{3+} after annealing at 1000 ˚C for 1 hour, compared with the XRD pattern of pure YVO_4 powder.[59]

58. G.; Schmidt Lohmüller, G.; Deppisch, B.; Gramlich, V.; Scheringer, C., *Acta Crystallogr. B*, 1973, **29**, 141-142.

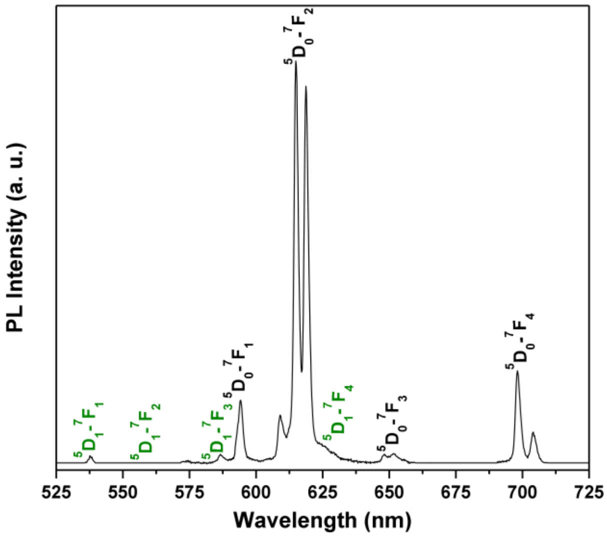

Figure S3 Emission spectrum of YVO_4: 2 p% Eu^{3+}.

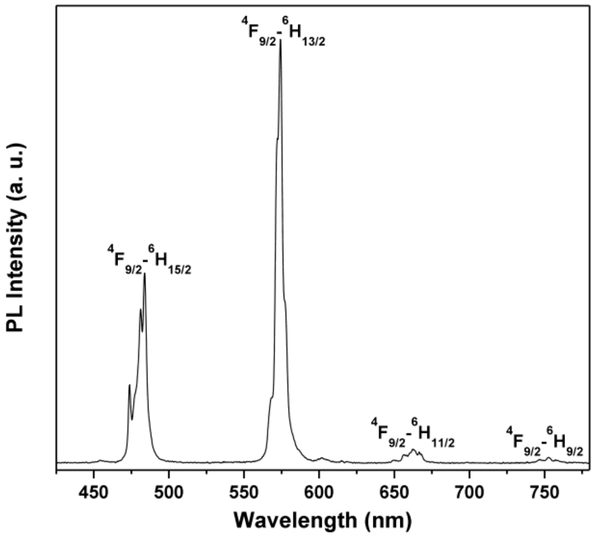

Figure S4 Emission spectrum of YVO_4: 2 p% Dy^{3+}.

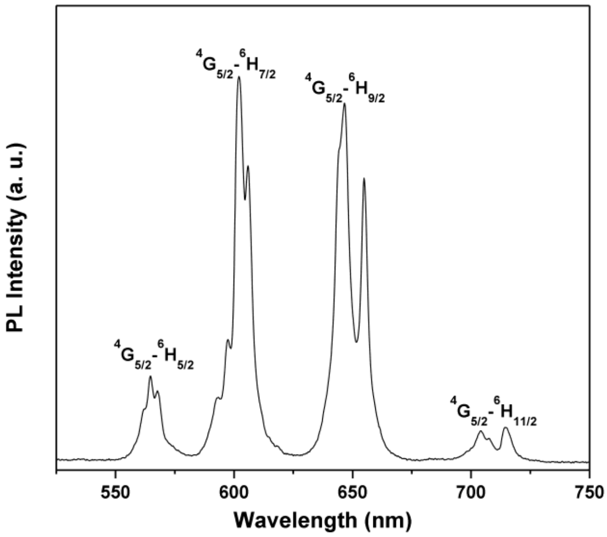

Figure S5 Emission spectrum of YVO_4: 2 p% Sm^{3+}.

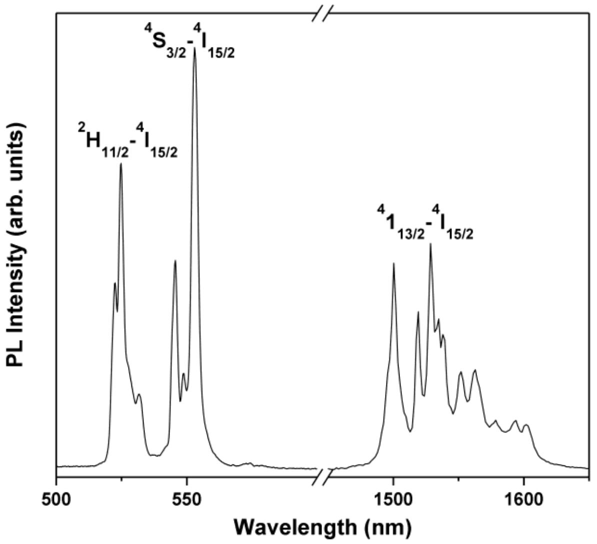

Figure S6 Emission spectrum of YVO_4: 2 p% Er^{3+}.

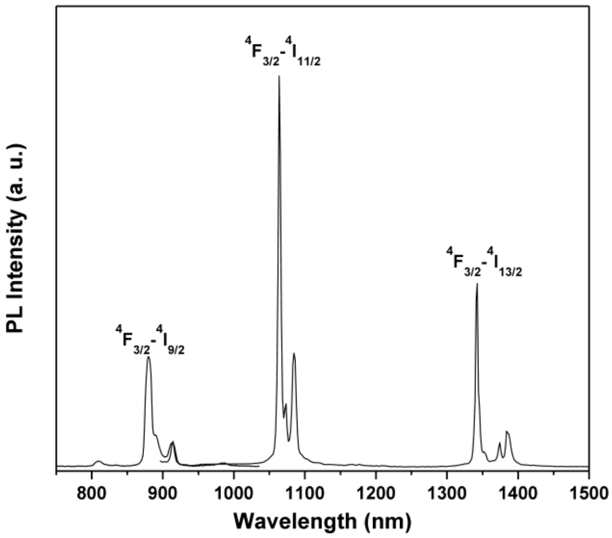

Figure S7 Emission spectrum of YVO_4: 2 p% Nd^{3+}.

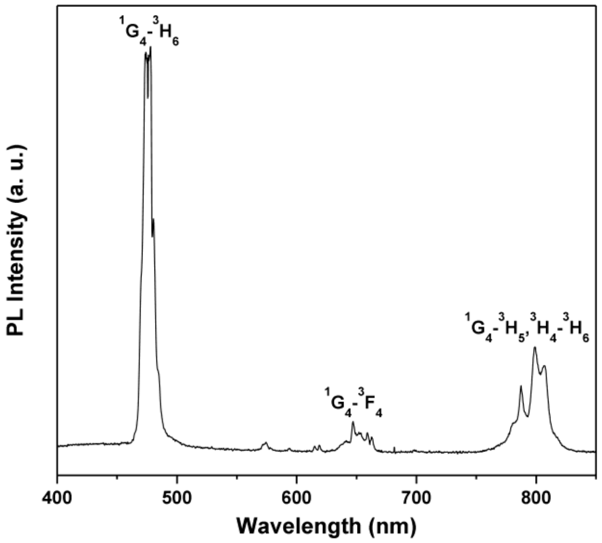

Figure S8 Emission spectrum of YVO_4: 2 p% Tm^{3+}.

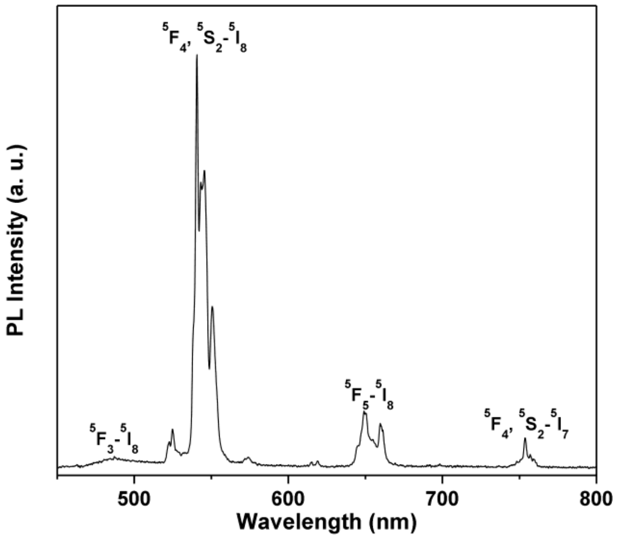

Figure S9 Emission spectrum of YVO_4: 2 p% Ho^{3+}.

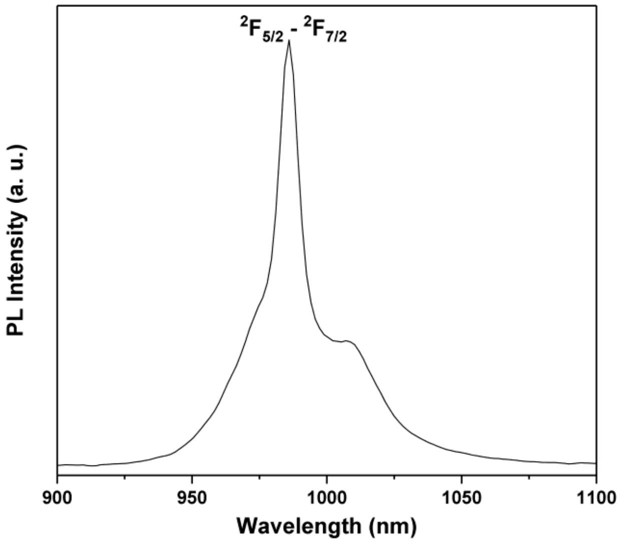

Figure S10 Emission spectrum of YVO_4: 2 p% Yb^{3+}.

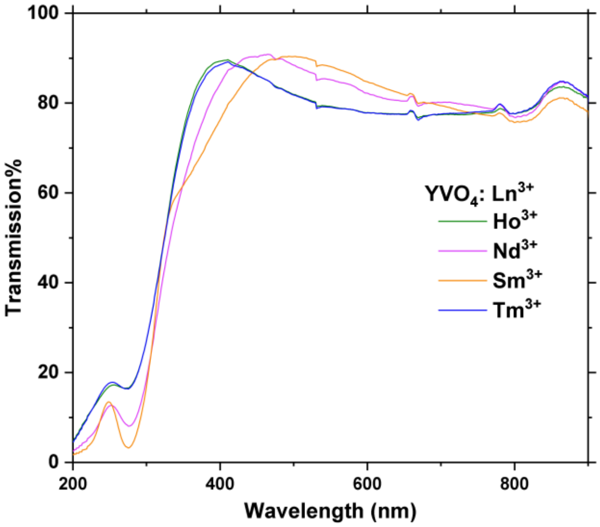

Figure S11 Transmission spectra of YVO_4: Ln^{3+} (Ln = Ho, Nd, Sm, Tm).

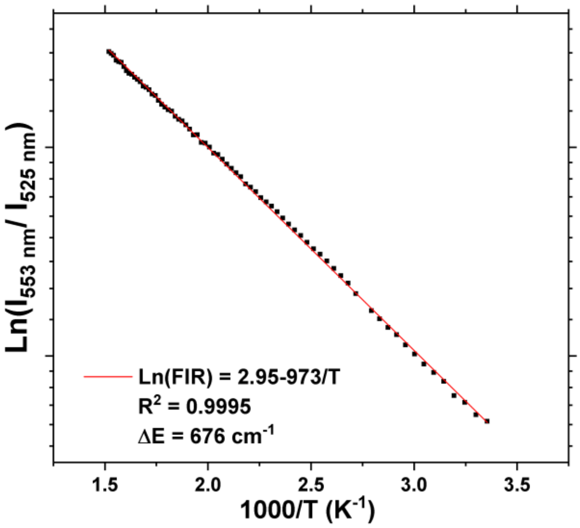

Figure S12 The thermal dependence of the FIR between the peaks at 525 nm ($^2H_{11/2} - {}^4I_{15/2}$) and 553 nm ($^4S_{3/2} - {}^4I_{15/2}$) of an YVO_4: 1p% Er^{3+} thin film, fitted to a linear function.

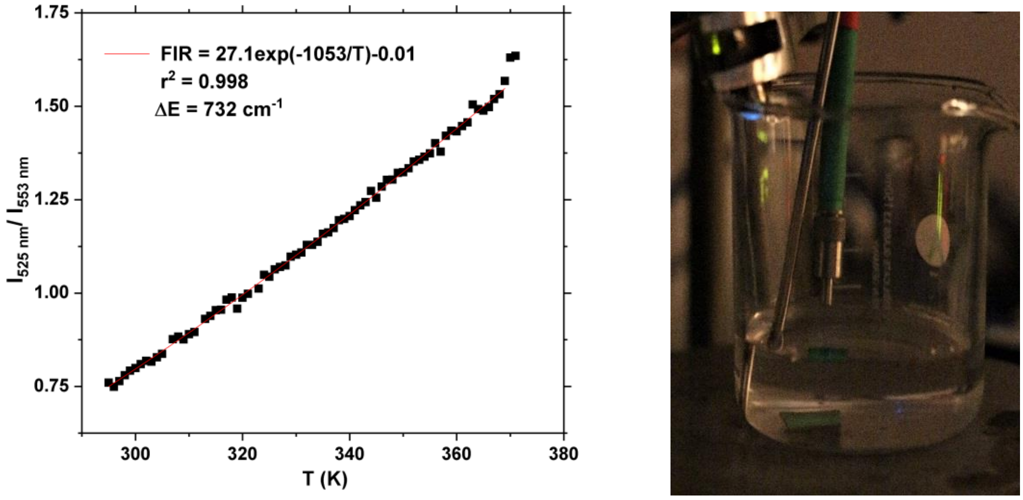

Figure S13 The thermal dependence of the FIR between the peaks at 525 nm ($^2H_{11/2} - {}^4I_{15/2}$) and 553 nm ($^4S_{3/2} - {}^4I_{15/2}$) of an YVO_4: 1p% Er^{3+} thin film, measured while the sample was submerged in water. The data points at 369-370 K were omitted from the fit due to the water starting to boil, causing the sample to move significantly. A picture of the setup is shown on the right.

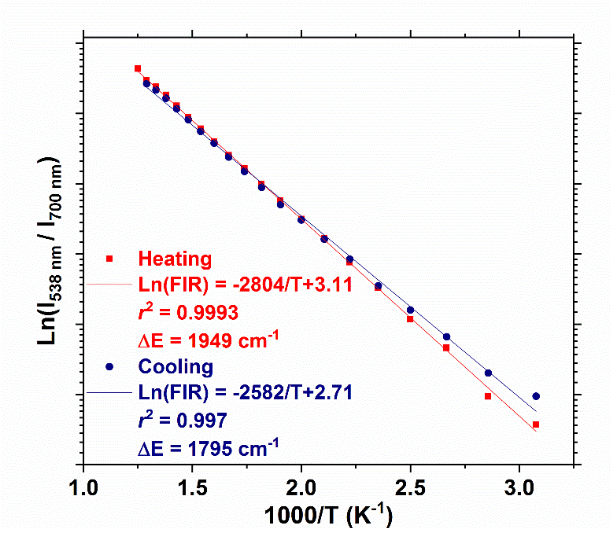

Figure S14 The thermal dependence of the FIR between the 538 nm ($^5D_1 - {}^7F_1$) and 700 nm ($^5D_0 - {}^7F_4$) emission of an YVO_4: 2p% Eu^{3+} thin film, fitted to a linear function.

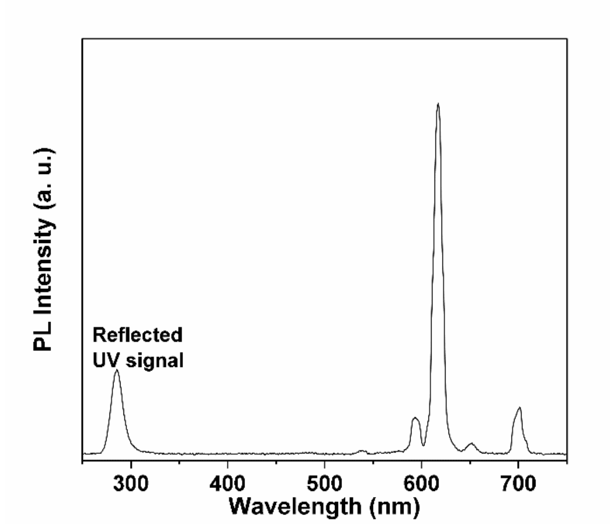

Figure S15 PL emission of an YVO_4: 2 p% Eu^{3+} thin film submerged in water. The signal in the 280 nm region is due to reflection from the UV light source.

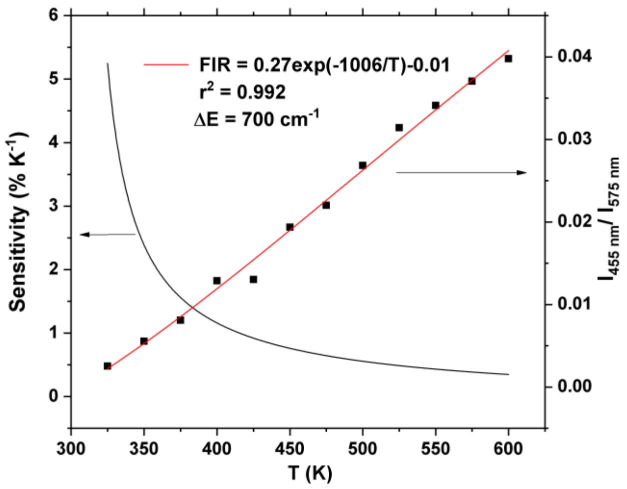

Figure S16 The thermal dependence of the FIR between the emission at 455 nm ($^4I_{15/2} - {}^6H_{15/2}$) and 575 nm ($^4F_{13/2} - {}^6H_{15/2}$) of Dy^{3+} in the dual layered YVO_4: Eu^{3+}/ YVO_4: Dy^{3+} thin film. The red line shows the best fit for the experimental data (black squares) to the equation: FIR = C exp($\Delta E\ k_b^{-1} T^{-1}$). The black solid line shows the corresponding relative temperature sensitivity.

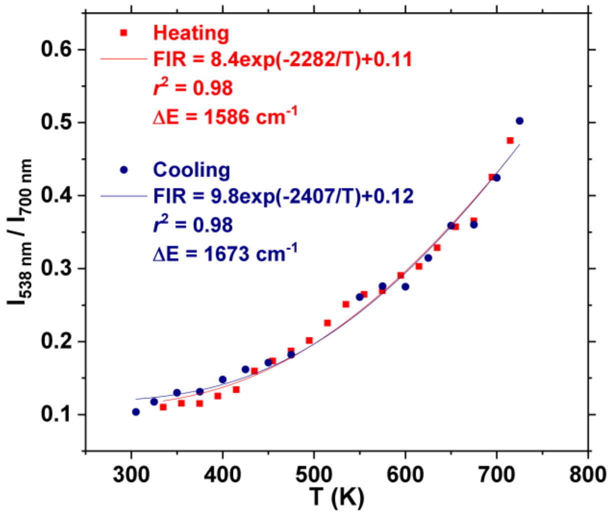

Figure S17 The thermal dependence of the FIR between the 538 nm ($^5D_1 - {}^7F_1$) and 700 nm ($^5D_0 - {}^7F_4$) emission in a dual layered YVO_4: Eu^{3+}/ YVO_4: Dy^{3+} thin film.

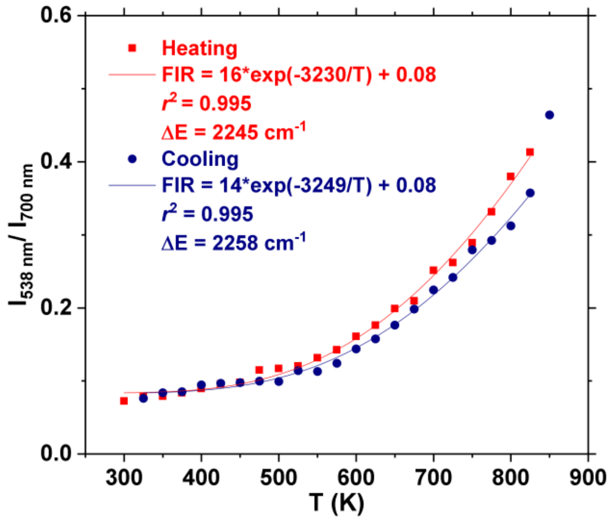

Figure S18 Ratiometric intensity of the $^5D_0 - {}^7F_4$ and the $^5D_1 - {}^7F_1$ transitions in a dual layered YVO_4: Tm^{3+}/ YVO_4: Eu^{3+} thin film.
